# Three Years on From “Stay at Home”: Perspectives of People With Intellectual Disabilities About the Ongoing Impact of the COVID‐19 Pandemic

**DOI:** 10.1111/jar.70076

**Published:** 2025-07-07

**Authors:** Jodie Rawles, Sue Caton, Dawn Cavanagh, Chris Hatton, Richard P. Hastings, the Coronavirus and People with Learning Disabilities Study Team, Richard P. Hastings, Chris Hatton, Jill Bradshaw, Sue Caton, Dawn Cavanagh, Amanda Gillooly, Andrew Jahoda, Rosemary Kelly, Roseann Maguire, Edward Oloidi, Jodie Rawles, Laurence Taggart, Stuart Todd

**Affiliations:** ^1^ Department of Social Care and Social Work Manchester Metropolitan University Manchester UK; ^2^ School of Social Policy and Society University of Birmingham Coventry UK

**Keywords:** COVID‐19, intellectual disabilities, learning disabilities, pandemic, qualitative

## Abstract

**Background:**

People with intellectual disabilities were disproportionately negatively affected during the COVID‐19 pandemic, but there has been limited research about the perceived longer‐term impact.

**Methods:**

Data were collected through a two‐stage narrative interview process with eight adults with intellectual disabilities. Participants told their storey about their lives at the time of the first lockdown in 2020, what their lives were like 3 years later, and what they hoped for the future. A second interview filled any gaps in the narrative. Data were analysed thematically.

**Results:**

Four themes were identified: navigating disruptions to meaningful activities; unique challenges associated with residing in group‐living environments; anger at the Government; barriers to moving forward.

**Conclusions:**

The themes identified reflect powerful emotional memories of challenging experiences during the COVID‐19 pandemic. Life continues to be difficult for many due to ongoing difficulties in getting the right support.

**Summary**

- The COVID‐19 pandemic made life very difficult for lots of people with intellectual disabilities.
- We wanted to hear from people with intellectual disabilities about their lives 3 years on from the start of the COVID‐19 pandemic.
- Eight people with intellectual disabilities in England did two interviews each with us. They told us about their lives during and after the pandemic.
- We found that:
  ○ People found it very difficult when their normal activities and routines changed.
  ○ People living in supported living and residential homes had extra rules to follow.
  ○ People are angry at how the Government acted during the pandemic.
  ○ Life now is hard for some people. They are finding it hard to get the support they need, and they worry about the cost of living.

## 1 Background

Whilst the COVID‐19 pandemic has significantly affected the lives of people across the world, people with disabilities (including those with intellectual disabilities) have been differentially affected (Shakespeare et al. 2021). Disabled people have faced a “triple jeopardy” (1311, *ibid*.), having been more likely to die or become seriously ill from COVID‐19 (Public Health England 2020; Sosenko et al. 2023), disproportionately impacted by reduced access to routine health care (House of Commons Women and Equalities Committee 2020; Flynn et al. 2021, 2022; Hatton et al. 2023, 2024), and further isolated and excluded from society by the measures taken to mitigate the pandemic (Clifford and Barratt 2022). The differential impact of COVID‐19 on the lives of people with intellectual disabilities in England was evidenced in the first few months of the pandemic, as during this time people with intellectual disabilities died from the virus at a rate six times higher than those without such disabilities (Public Health England 2020). Research since has consistently highlighted the ways that people with intellectual disabilities in England were negatively affected throughout the pandemic, including deteriorating mental and physical health, the closure of support services, and extended periods of separation from friends and family due to the additional guidance and restrictions that many people with intellectual disabilities were subject to (Flynn, Hayden, et al. 2021; Pearson et al. 2023; Scherer et al. 2023). Taggart et al. (2022) and others (see Keenan and Doody 2023) have highlighted how these impacts occurred in a context where public‐health policies and strategies were made without an awareness of the specific needs of people with intellectual disabilities nor the likely negative impacts of those policies.

In addition to the research evidence highlighting negative impacts of pandemic protections, positive changes associated with the pandemic have also been noted. Most significantly, there has been a consistent narrative of the buffering impact of online communications during times of restrictions (Casey et al. 2023) with people enjoying online social connections (Caton et al. 2022) and digital inclusion facilitating a positive effect on wellbeing (Chadwick et al. 2022). Other positive impacts of the pandemic have also been identified, with some paid carers reporting fewer demands on time (Keenan and Doody 2023), increased opportunities for trying new activities, and subsequent gains in independence and decision‐making skills (Casey et al. 2023).

To date, research has provided valuable insights into the experiences of people with intellectual disabilities in the U.K. during the early months of the COVID‐19 pandemic. Such research is based on data collected around this time. For example, the work of Pearson and colleagues (Pearson et al. 2023; Scherer et al. 2023; Shakespeare et al. 2022) is based on the interviews they conducted with people with intellectual disabilities and other key informants between June and August 2020. However, the medium‐to long‐term effects of the pandemic on the lives of people with intellectual disabilities remain unclear (Keenan and Doody 2023). There are indications from survey research and other reports that people with intellectual disabilities are still facing issues exacerbated by the pandemic, such as the “cost‐of‐living crisis” and the recruitment and retention difficulties in adult social care (Coronavirus and People with Learning Disabilities Study Team 2022; Hatton et al. 2023; Health and Social Care Committee 2022). However, missing from the literature are in‐depth accounts of people with intellectual disabilities reflecting on what turned out to be a significant period of restrictions, and how this period has, if at all, impacted their current lives and visions for the future. Such accounts are important for understanding current needs and for informing responses to future crises.

The current study sought to elicit the accounts of people with intellectual disabilities reflecting on their lives since the first national lockdown in England was announced in March 2020. Narrative interviews were carried out with people with intellectual disabilities approximately 3 years after the nation was first instructed to “stay at home” (Prime Minister's Office 2020). This study aimed to examine the storeys people with intellectual disabilities told about what living through the COVID‐19 pandemic meant, what impact this had on their daily lives, and on their plans and hopes for the future.

## 2 Materials and Methods

### 2.1 Research Approach

This research was carried out using what Polkinghorne (1995) terms, the “analysis of narratives” (12) approach. This involves using a narrative approach to elicit storeys from multiple individuals which are then analysed thematically. A narrative approach holds that people construct storeys to make sense of their experiences, including the disruptions they experience in life (Becker 1997; Murray 2015). This approach was therefore deemed particularly suitable for examining how people with intellectual disabilities made sense of the great disruptor that was the COVID‐19 pandemic, alongside their present lives. By analysing data thematically, we sought to identify similarities and differences in the storeys told by a group of people with intellectual disabilities with different characteristics and circumstances.

The study was grounded in a critical realist perspective. Critical realism posits that an objective reality exists independent of our subjective experience or knowledge of it (Danermark et al. 2002). This perspective recognises that the researchers and participants were operating upon a shared understanding of reality and timeline of events during the period of interest, whilst holding space for individuals' unique experiences.

### 2.2 Participants and Sampling

The inclusion criteria were that the persons identified as having an intellectual disability, they were 16 years or older, were able to take part in an interview by phone or video with or without support, and had previously taken part in [study redacted for peer review]. Using our database of participants from the wider study, purposive sampling was used to recruit people with different characteristics and circumstances, such as those with and without paid work, those with different living and support situations, those from diverse ethnic communities, and people from different age groups. The diversity of the sample was monitored throughout a gradual recruitment process, so that attempts could be made to recruit individuals who possessed characteristics or had circumstances not already represented in the sample. The rationale behind this broad sample frame was the exploratory nature of the study. It was the first, to the best of our knowledge, to interview people with intellectual disabilities about the COVID‐19 pandemic, at a time‐point when associated legal restrictions were no longer in place. As such, we felt there was insufficient rationale for narrowing in on the experiences of individuals who share a particular characteristic or circumstance. The only exception to this was the experiences of people with profound and multiple intellectual disabilities, as there is evidence that this group experienced more adverse outcomes because of the pandemic than those with milder disabilities (Hatton et al. 2023, 2024; Linden et al. 2022). In recognition of this, the caregivers of people with profound and multiple disabilities were interviewed as part of the wider project and the findings from this work are presented elsewhere [reference redacted for peer review].

Demographic data are reported here at the aggregate level to preserve anonymity. Eight people participated in this study. Six people were between 25 and 60 years old, one person was under 25, and one person was over 60. One person described themselves as Black and another person described themselves as Asian. The remaining six participants described themselves as White. Interviewees described having a range of support and living arrangements, including living at home with their partner or family (*n* = 3), living in supported living (*n* = 2), and living in a residential home (*n* = 3). In the U.K., residential homes refer to communal living environments where residents have their own bedrooms and support workers are available 24 h a day. In comparison, those in supported living are encouraged to exercise a greater level of independence and have their own individual tenancies. Seven of the eight participants reported receiving social care support, which ranged from 24 h‐support to few hours per week. All but one person described being in paid (*n* = 5) or voluntary work (*n =* 2).

### 2.3 Procedure

Potential interviewees (who had taken part in the wider study) were approached about participating in the study using their preferred way to communicate with the researchers, as established from their involvement in previous parts of the wider research project (i.e., by telephone, email or through a supporter). Individuals interested in taking part were sent an easy‐read information sheet about the study and were informed that they would be offered a £20 shopping voucher to thank them for their time. Initial short, informal meetings were held with those wishing to proceed to discuss any questions potential interviewees may have had about the study. Potential interviewees were given various ways to contact to cancel the appointment and say they do not want to take part.

The study used a two‐stage interview process which took place between November 2022 and March 2023 using Zoom or Microsoft Teams. All interviews were conducted by X1 (first author name withheld for peer review) or X2 (third author name withheld for peer review), both skilled interviewers with experience of conducting research interviews with people with intellectual disabilities. Participants were always interviewed by the same interviewer. Each interview took between 30 and 90 min to complete. At the first interview, interviewees were invited to tell their storey of the last few years since the start of the COVID‐19 pandemic. Emphasis was placed on affording the interviewee as much freedom as possible in shaping and sharing their narrative. However, in most cases, the following prompts were used – “What was life like at the time of the first lockdown?”; “What is your life like now?”; and “What do you hope life will be like in the future?”. After the first interview, X1 and X2 individually reviewed all interview recordings and noted any chronological gaps in the narrative or uncertainties that would benefit from further exploration in the second interview. The notes of X1 and X2 were then combined to generate a unique semi‐structured guide for the interviewee's second interview, which took place approximately 1–2 weeks after the first. Common questions included those about the future, as time had often run out to cover this in the first interview, and clarification regarding the sequence or details of events. At the end of the second interview, interviewees were debriefed regarding the next steps of the study and, where appropriate, signposted to support services.

### 2.4 Analysis

The data was analysed using thematic analysis. Thematic analysis is a suitable method for analysing narrative texts when the primary interest lies in the narrative's content rather than its structure (Riessman 2008). Another advantage of thematic analysis is that it can be applied flexibly. As such, whilst the analysis was guided by the writings of Braun and Clarke (2022), minor adjustments to traditional procedures were made, as identified in the text that follows.

The analysis was led by X1. Audio recordings of interviews were transcribed by professional transcribers. Following familiarisation (achieved through listening to recordings, reading transcripts, and memoing), the data were coded by X1. To assess the reliability of X1's coding, X2 also coded several transcripts and the codes were compared to those identified by X1. There was a high level of inter‐coder agreement. Initial themes were first developed for each individual. Codes relating to each participant were reviewed and organised into candidate themes which captured the person's narrative. This differs from a more traditional approach to thematic analysis, such as that articulated in Braun and Clarke (2022), whereby themes are developed by reviewing codes across the sample. It was felt that this approach would decontextualise the codes too early in the analytic process and risk severing the threads in the individual's narrative. Once themes were established on an individual basis, comparisons were made between accounts. A working thematic framework was then developed to reflect the sample as a whole. Iterations of the final thematic framework were developed through comparison with the original data set, writing up, and discussion with the wider research team, until the team was satisfied that the framework accurately represented the storeys told by participants.

### 2.5 Consent and Ethical Considerations

Research ethics approval was sought and obtained from the [details removed for peer review] Ethics Committee. As part of the informal meeting that took place prior to the interviews, the information sheet was reviewed with interviewees to ensure they understood its contents. A standardised script in straightforward language that was issued by the University for use in online interviews during the pandemic was used to obtain consent from interviewees before the first interview began. Consent was audio recorded rather than written to avoid placing additional technological and time demands on interviewees. Interviewees were reminded of the core principles of consent before the second interview commenced (e.g., that they could withdraw from the study if they wished and they did not need to talk about anything that made them feel uncomfortable). The audio files documenting consent were stored securely and separately from recordings and transcripts. Pseudonyms have been allocated to interviewees to preserve anonymity.

## 3 Results

The results are presented here as four themes which together capture what living through the COVID‐19 pandemic meant to the interviewees, and the impact of these experiences on their current lives and concerns for the future. Table 1 below sets out these themes and their subthemes.

**TABLE 1:** Themes and subthemes.

Theme	Subtheme
1. Navigating disruption to meaningful activities	1(a) Disruption to meaningful activities 1(b) Negative impacts of disruption on mental and physical health 1(c) The role of others in providing access to social and leisure activities
2. Unique challenges associated with residing in group‐living environments	2(a) Challenges around social distancing and isolation 2(b) Disparities in restrictions 2(c) Bereavements in the home
3. Anger towards the Government	
4. Barriers to moving forward	4(a) Difficulties securing the right support 4(b) Concerns around rising costs

Table 2 below provides a list of interviewees' pseudonyms and their living situations to aid the reader's understanding of the narratives.

**TABLE 2:** Participants' pseudonyms and reported support and living arrangements.

Pseudonym	Support and living arrangements
Brandon	Residential home with some time spent living with parents
David	Residential home
Edna	Residential home
Sarah	Supported living
Victoria	Supported living with some time spent living with parents
Eric	Living with family, no paid support
Sam	Living with family, some paid support
Steven	Living with partner, paid support began after onset of pandemic

### 3.1 Theme 1: Navigating Disruption to Meaningful Activities

I spent a couple of days crying, constantly crying because I was frustrated about not being able to do things I was capable of doing.Sarah

Theme 1 highlights the impact that the disruption to usual activities had on people's lives in terms of mental and physical health and a shifting reliance on families and organisations from the voluntary sector.

#### 3.1.1 Subtheme 1(a): Disruption to Meaningful Activities

When looking back and describing what life was like during times of COVID‐19 related restrictions, interviewees emphasised the disruption to the meaningful activities in their life. The term meaningful activities is used here to describe the social and leisure pastimes that interviewees described engaging in, as well as daily acts of self‐care, such as shopping for groceries or picking up medications.

All the interviewees talked about their pre‐pandemic lives where they engaged in a range of activities and social relationships they drew pleasure from. These activities included paid and voluntary work, sport, participation in self‐advocacy groups, and involvement in higher education teaching and research. Inevitably, such activities were disrupted by the pandemic. When speaking about their lives during COVID‐19 restrictions, many of the interviewees appeared to focus on this disruption and the different things they were unable to do. There was little discussion of alternative pursuits. Rather, most interviewees repeatedly emphasised that they were not “allowed” to do “anything” or see “anyone”, communicating a sense of all access to meaningful activities and relationships being stripped away. In Eric's words, “…you felt like you were stuck in all the time. You couldn't do anything, you couldn't go anywhere, you couldn't see anyone”.

In addition to social and leisure activities, interviewees also expressed dissatisfaction at being prevented from carrying out activities of daily living that they would normally do themselves. For example, Edna recalled being “upset” because, rather than going out with staff to do her “personal shopping”, Edna had to “write out a shopping list and give it to the staff to shop for [her]”. Similarly, Sarah recalled “being really really scared” about being unable to collect her prescription as normal when she was required to isolate. She explained:

**Excerpt 1**

… I'm so used to being able to walk down to the chemist [pharmacy], go, pay for my medication, because I work, and then come back up the hill, back to my flat and then sorted. But I was really worried about not being able to get out, to be allowed out of my flat to go and get my medication. I was absolutely so frustrated about not getting my medication…Sarah

Whilst it may be considered an inconvenience by some, for Sarah, collecting her prescription seemed to be a valuable source of routine and potentially pride, given that Sarah highlighted that she pays for her medication herself as she works. Being unable to pick up her medication would threaten this routine and sense of pride, leaving Sarah feeling “scared” and “frustrated”.

#### 3.1.2 Subtheme 1(b): Negative Impacts of Disruption on Mental and Physical Health

The interviewees identified various ways their mental and physical health was negatively impacted by the disruption to their usual meaningful activities. A few interviewees gave examples of the ways their physical health had been impacted, such as disruption to sleep and weight gain. However, interviewees mainly spoke about the impact on their mental health. For example, Sarah described how, upon the first national lockdown, she spent “a couple of days in [her] room crying” as she was frustrated about being unable to “go and do things [she] was capable of doing”. Eric who lived at home with his family, spoke about what the lockdown meant for him:

**Excerpt 2**

[…] during the lockdown I was at home, I was bored, I was depressed. I was struggling to just maintain my physical and mental health. It was really challenging. And I felt like all the activities, all the social life I had, that was taken away from me. And […] I was really, really, really heartbroken.Eric

Like Eric, Steven also described lockdown as an extremely difficult time. Steven lived in an apartment with his partner who he described as having intellectual and physical disabilities. During the national lockdowns, Steven described how he and his partner lived in a small apartment and had limited space. Normally a very active person who spends a lot of time outside of his flat, Steven had little to do, his only pastimes being “listening to music and watching Youtube”. Steven recounted how he found this boredom, combined with a lack of space and caring for his partner, “too hard” and that he was “frighten[ed]”. Steven explained that eventually he had a “breakdown”, which culminated in a period of separation between him and his partner. In summary, it appeared that all the interviewees struggled to some extent with their usual activities and routines being disrupted.

#### 3.1.3 Subtheme 1(c): The Role of Others in Providing Access to Social and Leisure Activities

An important function family members and charitable organisations appeared to play during the pandemic was re‐establishing access to social and leisure activities. This is evident in the account provided by Victoria, who was the only individual to foreground all the activities she did during times of restrictions rather than the things she was unable to do. These activities appeared predominately orchestrated by her family. One of the activities that “sticks in [Victoria's] mind” was a Christmas advent calendar of “treats”, “jokes” and “activities”, delivered to her in supported living by her family.

Many interviewees shared their gratitude to charitable organisations, including self‐advocacy, creative, and other social groups, for hosting meetings and other activities on video‐conferencing platforms such as Zoom, as well as providing access to the digital devices required to join the calls. Whilst some acknowledged that virtual meetings did not fully replace face‐to‐face contact, for certain interviewees, the calls were one of the only pastimes they had. As David, who described himself as being “really down” during restrictions put it, “without [organisation's] Zoom calls, I don't know where I'd be”. In this way, charitable organisations drew upon the benefits of technology to provide interviewees with a valuable sense of meaning and connection that they were otherwise bereft of as a result of COVID‐19‐related restrictions.

### 3.2 Theme 2: Unique Challenges Associated With Residing in Group‐Living Environments

…They're coming in and bringing us the COVID, and we're not allowed to even have a walk.Brandon

Interviewees living in group‐living environments, defined here to include supported living and residential homes, described experiencing challenges that those living with family or a partner did not recount. As will be illustrated below, these challenges appeared to be a product of the restrictions interviewees faced, combined with the complexities of residing in close proximity with multiple people with intellectual disabilities. Interviewees described how these challenges gave rise to feelings of frustration, fear, and injustice. Interviewees identified such feelings explicitly, as well as communicated them more implicitly in the scenes they described.

#### 3.2.1 Subtheme 2(a): Challenges Around Social Distancing and Isolation

One of the first restrictions David and Edna reported experiencing was that they were denied access to communal spaces in the home, which made them feel frustrated. One of Edna's earliest memories of the pandemic was her and other residents “getting worked up and into a state” as they were prevented from doing their usual group activities in various parts of the residential home. Meanwhile, David recalled how he “shouted quite a few times” as initially he could not understand why he was no longer permitted to watch television in the communal space. Those not living in group‐living environments did not describe having their movements around the home restricted in this way.

Based on participants' accounts, it appears that when residents in group‐living environments were permitted to share communal spaces, some providers tried to encourage social distancing, defined as approximately 2 m apart (Public Health England 2021). Again, this is not a practise those living with family described having to do. Several participants in group‐living environments identified that they or those around them found it difficult to maintain their distance as they struggled to understand why it was important to do so. This seemed to create anxiety and frustration in the home. For example, Edna recalled repeatedly “shouting” at her housemate, who failed to keep her distance, to “go away”.

Whilst none of the participants residing with family or their partner described having to self‐isolate, four of the five participants who resided in group‐living environments described having to on at least one occasion. Self‐isolation means not having contact with others and in households with multiple residents, such as group‐living environments, may involve the person residing predominately in one room (Public Health England 2021, 2022). For those living in residential homes, this meant self‐isolating in their bedrooms. Brandon was very anxious about this prospect, as he worried that it would trigger memories of being placed in seclusion in hospital. Whilst fortunately Brandon did not need to isolate, Sarah, Victoria, Edna and David did. David described this as a particularly distressing time for various reasons. Whilst Sarah, Victoria and Edna recalled isolating for approximately one to two weeks, David described being confined to his room for “six to eight weeks”. David described living in a residential home housing people with additional respiratory and cardiac issues. David recalled that COVID‐19 “went all round the place” and that his home entered a “lockdown” that they were not able to come out of until approval was given by the local hospital and county council. David described getting “square eyes” from “watching TV all the time” as this was all he could do. He described getting “really down”. A practise adopted by the home which appeared to negatively impact David's mental wellbeing further, was their procedure for delivering meals. In the interests of infection control, staff left residents' meals on their bedroom floors. David, who described himself as having difficulties with balance and mobility due to physical disabilities, said it was extremely challenging for him to pick up the meal trays and that, as a result, he had “quite a few accidents” with “dinners thrown on the floor”. David's frustration and sense of injustice at how he was being treated can be seen in Excerpt 3 below.

**Excerpt 3**

…when I lifted [the meal tray] up, I couldn't lift it up properly. So I ended up spilling it. So I ended up pressing the buzzer [to receive help]. They weren't happy. Then a few weeks later I went down to the boss. I said “Why are you doing this?”Infection. To do with the infection.I said to them, “Can't you see I've got mobility problems? You should know that. You've known me for God knows how long. You can't do that with me.” I went mad at them.David

Perhaps reflecting the emotional salience of the memory, David chose to open his storey of the last few years by talking about this time period in which he was confined to his room for “weeks on end” and delivered meals that he struggled to pick up.

#### 3.2.2 Subtheme 2(b): Disparities in Restrictions

In addition to the injustice around how David was treated, Brandon also communicated a sense of injustice in highlighting disparities in the restrictions that individuals in different group‐living environments were subject to. For example, Brandon noted that whilst his “friends in supported living” were “doing stuff”, the lockdown in residential homes “was very very severe”. Indeed, Sarah, who resided in supported‐living, described going for walks with other tenants—a practise Brandon or David were not permitted to do. Additionally, Brandon noted how staff were not subject to the same restrictions as residential home residents, who were not permitted to leave the premises. As evident in Excerpt 4 below, Brandon recounted how the staff flaunted their relative freedom and disregard for “the COVID rules”. Brandon suggested that such behaviour had led to people he knew living in residential homes testing positive for COVID‐19 on multiple occasions despite not leaving their homes.

**Excerpt 4**

But, of course, whilst we we're not allowed to do anything, [support staff] were always coming saying, “Oh, we've [had a] massive party, 100 of us, you know, screw the COVID rules […] we don't care. We didn't wear masks.” And yet they're coming in bringing us the COVID, and we're not allowed to even have a walk.Brandon

#### 3.2.3 Subtheme 2(c): Bereavements in the Home

For two interviewees residing in group homes, the last few years also appeared marked by the emotional impact of a housemate becoming unwell with, and subsequently dying from, COVID‐19. Brandon described how his housemate, who struggled to comply with social distancing and isolation requirements, became very unwell and was subsequently admitted to hospital where they later died. Whilst it is well documented that a disproportionate number of people living in group‐living environments died from COVID‐19 (Tessier et al. 2023), inevitably many people living outside of these environments will have experienced someone they live with becoming unwell and sadly passing away from COVID‐19. Nonetheless, it appears from Brandon's description that being in a group‐home added further emotional complexities to this experience. Due to COVID‐19 tests not being available in residential homes at the time and “rules about confidentiality” (Brandon), the residents were not informed whether the housemate's illness was attributable to COVID‐19 till after their death. For Brandon, this uncertainty created additional anxiety and panic within the home:

**Excerpt 5**

Like we're worried for [unwell housemate] and also slightly… […], sort of selfish side […], we were kind of terrified as well because […] nobody's telling you stuff […] and their rules about confidentiality […] they're not allowed to tell you other people's business. But also […] being locked in a house with somebody who's really sick, and all that you're hearing on the news is COVID and everybody freaking out and parents wanting to know and everybody scared and them saying, “Oh, we can't tell you anything.” And you know, God, are we all about to die of COVID? […] It was very scary.Brandon

David also experienced the bereavement of a housemate due to COVID‐19. David became upset when this subject arose during an interview and chose not to share any additional details. David was supported by the interviewer during the interview and by his self‐advocacy organisation after its conclusion. As David's wish not to discuss the bereavement further was respected by the interviewer, it is unclear whether David received any additional support at the time of the bereavement.

### 3.3 Theme 3: Anger Towards the Government

The Government just don't even have a clue about what the pandemic has done to people like me, with an [intellectual] disability.Eric

In providing their storeys of the last few years, seven of the eight interviewees referenced the Government's handling of the pandemic. Sam and Eric communicated a palpable sense of anger, particularly in relation to the “lockdown parties” (Eric)—a series of gatherings on Government premises that violated regulations and guidance at the time (Gray 2022). Both Eric and Sam perceived these gatherings as evidence that the Government did not “care” about them or the public. As seen in Excerpt 6, Sam was particularly angered by these parties given that their mum was undergoing treatment for cancer at the time:

**Excerpt 6**

Sam: I was really, really angry when they had a party, 10 Downing Street, I was really, really angry with it because people's loved ones were dying and they didn't really care. […] People were losing their loved ones, people were losing their daughters, their kids […] And the government didn't really… weren't acknowledging that. They were throwing parties for themselves.

Interviewer: And what was going on for your family at the time?

Sam: My family was going through a lot because we were going through cancer, and the surgeries and stuff like that. […] I saw a woman on the ward who passed away with COVID and it just brought me to tears.

For Eric, the gatherings left him feeling “let down” and “betrayed”, as he had given up “two years of [his] freedom, two years of [his] social life”. In addition to the gatherings, several interviewees suggested that the Government had failed to consider the impact of COVID‐19‐related measures on people with intellectual disabilities. Eric felt that the Government was ignorant of “what the pandemic has done to people like [him], with a [intellectual] disability”. As a result of their actions during the pandemic, Eric reported that he no longer had “respect for the Government” and that he will “never forgive them for what they've done”. However, Steven and Sarah said they hoped that the Government could “learn” from the pandemic, that in the future, steps would be taken to avoid further lockdowns and, if lockdowns were necessary, additional arrangements would be made to ensure people with intellectual disabilities could access support.

### 3.4 Theme 4: Barriers to Moving Forward

…with the prices of living going up, how is it going to affect us? If the prices are going up […] what are we going to do with our flat and everything like that? […] are we going to be on the street?Steven

Interviewees were mixed in their feelings about the future. Some people seemed optimistic about a future less impacted by COVID‐19 restrictions. For example, Eric described how he wanted “to move forward to the future, move onto bigger and better things”, such as working more and using the money to go on holiday. However, interviewees also described difficulties they had encountered in re‐establishing a life with adequate support and voiced concerns regarding rising costs.

#### 3.4.1 Subtheme 4(a): Difficulties Securing the Right Support

Brandon and Steven spoke at length about barriers they encounter in securing the support they need. Whilst COVID‐19 restrictions were in place, Steven described having a “breakdown”, whilst numerous incidents had highlighted that Brandon's support arrangements were not adequate to keep him safe. Brandon and Steven described how, whilst these events had initially triggered changes to be made to their housing and/or support, they were continuing to encounter barriers to securing the right arrangements and that their wellbeing was being compromised as a result. For example, Steven largely attributed his “breakdown” to feeling overwhelmed by his caring responsibilities for his partner and domestic chores. Consequently, Steven was offered support for domestic chores, but he described how, many months later, the support remained inconsistent due to staff shortages. Steven had therefore taken up more chores again, with his stress rising as a result.

#### 3.4.2 Subtheme 4(b): Concerns Around Rising Costs

Half of the interviewees observed that the cost of essentials such as groceries and heating was rising and expressed anxiety around this. For example, in addition to his worries about getting the support he needs, Steven feared being unable to afford his rent, as illustrated in the above quotation used to introduce Theme 4. Sarah described taking her financial worries to her “lead support worker”, and that together, they worked out Sarah's expenses. In Sarah's words, she “showed me just how hard it is at the moment. And believe you me, it is tough, very tough at the moment […] everyone has got to tighten their belts”. Some interviewees also discussed how rising costs had affected them resuming access to the activities they enjoyed. Both Sarah and Sam reported that they attended their usual social groups less because the prices to attend them had risen. Some interviewees described how local services and facilities had been withdrawn or were under threat. For example, Sam, who enjoys swimming, said they were concerned that they had heard their local pool may shut due to heating costs, whilst Steven, who uses the bus to get around, said he worried about proposed cuts to local bus routes.

## 4 Discussion

This research contributes an analysis of the storeys told by eight people with intellectual disabilities in England when asked to look back on their experiences approximately 3 years after the nation was first instructed to “stay at home”. Existing research on the impact of the COVID‐19 pandemic on people with intellectual disabilities draws on data collected in 2020 and 2021 whilst restrictions were still in place. This study therefore provides unique insights into what, given the passage of time, stands out to people with intellectual disabilities about their experiences during the pandemic, where they are at now, and their priorities moving forward. This is important knowledge for responding to the current needs of people with intellectual disabilities and informing responses to future crises.

The interviewees characterised the pandemic as a time when all their meaningful activities were stripped away, which negatively impacted their mental health. Similar reports were provided by the people with intellectual disabilities interviewed in the Netherlands (Voermans et al. 2022) and the United States (Randall and McKown 2022) during the pandemic. Both Voermans et al. (2022) and Randall and McKown (2022) argue that people with intellectual disabilities experienced disruptions to their usual activities more keenly than the general population because restrictions placed further constraints on their already limited opportunities for living self‐determined lives. The emphasis interviewees placed on what they were unable to do over alternative pursuits may also reflect the difficulties that people with intellectual disabilities can face in accessing the resources (e.g., financial, social, technological) required to establish new pastimes (Merrells et al. 2019; Mooney et al. 2019). Indeed, the findings from this study highlighted the importance of family and charitable organisations in facilitating access to alternative meaningful pastimes and connections, often through technology. This echoes the findings of Cullingworth et al. (2022) who found that third‐sector organisations played an essential role in providing people with access to digital devices and running online events. Whilst the findings of this study lend further support to reports that people with intellectual disabilities enjoyed online connections during the pandemic (Caton et al. 2022), it is unclear to what extent this would apply to people with intellectual disabilities with lower levels of digital literacy and access (Chadwick et al. 2022).

The storeys told by interviewees made clear that they felt the pandemic had had a significant negative impact on their mental, and in a few cases, physical health, supporting the findings of previous qualitative (Clifford and Barratt 2022) and quantitative (Flynn et al. 2021) research conducted in the U.K. and internationally (Lunsky et al. 2022). What is less clear, in this study and more broadly, is to what extent the acute psychological distress experienced by people with intellectual disabilities during restrictions has persisted and/or evolved into a mental health condition. The vividity with which, 3 years on, interviewees recalled distressing instances that occurred during times of restrictions may indicate that these memories are traumatic, and that they may be accompanied by other trauma‐related symptoms (Porter and Peace 2007). However, the ability of interviewees to share their experiences with such vividity may also be a result of their ongoing participation in the wider, longitudinal project that this study formed part of, which required them to reflect on their experiences throughout the pandemic. Regardless, establishing the long‐term psychological impact of the pandemic on people with intellectual disabilities seems particularly pertinent given the higher prevalence of mental health conditions in this population (Perera et al. 2020) and the increased barriers they face in accessing appropriate treatment (Whittle et al. 2018).

As part of this investigation into the long‐term mental health impacts of the pandemic, the findings suggest that special consideration should be given to people with intellectual disabilities who reside in group‐living environments. The findings indicate that people in these environments experienced unique challenges, such as those associated with additional restrictions and bereavements within the home. Given the disproportionate number of deaths from COVID‐19 in people with intellectual disabilities (Public Health England 2020), particularly those in residential homes (Tessier et al. 2023), it is likely that there are many people with intellectual disabilities who, like Brandon and David, experienced the death of someone they were living with from COVID‐19. Given this, as well as the increased propensity of people with intellectual disabilities to experience “complicated grief” (p.833, O'Riordan et al. 2022), a worthy avenue of future research may be to investigate the impact of such deaths on people with intellectual disabilities who witnessed them and the support, if any, that people are accessing.

In light of their experiences during the pandemic, the interviewees expressed anger towards the Government regarding their actions and decision‐making during this time. Indeed, research has reported lower levels of trust in the government amongst disabled people in the UK compared to non‐disabled people during the COVID‐19 pandemic (Emerson et al. 2022). Reparative steps that could be taken include ensuring that the voices of people with intellectual disabilities are included in measures the Government and other bodies are taking, including the commission of the U.K. COVID‐Inquiry, to learn lessons from the pandemic. The findings of this study suggest that this is particularly important in relation to group‐living environments where decisions were taken at multiple levels, stretching from the highest level of Government all the way through to individual providers, which shaped the restrictions residents were subject to and had profound impacts on their lives. Government and other key decision‐makers should also be aware that, in addition to the difficulties they faced during the pandemic, the interviewees' storeys indicated significant concern regarding current societal issues including the cost of living and difficulties accessing social care. This supports the findings of other research that has found people with disabilities are being disproportionately impacted by the rising cost of living (El Dessouky and McCurdy 2023; Scope 2022), and reports that social care is in a “sustained state of crisis” (4, Hft and Care England 2024; Care Quality Commission 2023). As society seeks to combat these issues, there is an opportunity to take into account existing health and social inequalities, including those experienced by people with intellectual disabilities, which failed to adequately occur in the COVID‐19 response (Curry et al. 2023).

## 5 Strengths and Limitations

The aim of this study was, 3 years on from its commencement, to examine the storeys people with intellectual disabilities told about what living through the COVID‐19 pandemic meant, what impact this had on their daily lives, and on their plans and hopes for the future. Whilst interviewees did talk about both the future in terms of both getting back to normal and concerns about staff shortages and the cost of living, interviewees did tend to focus their narratives and responses around the powerful emotional memories that they were left living with as the UK began to move away from pandemic restrictions. Rather than viewing the relative lack of attention to the future as a limitation of this study, the focus on the past may be considered an important indicator of the legacy the pandemic holds in the minds of these people with intellectual disabilities.

The decision to carry out two interviews with each participant and to allow them to prepare by sharing the interview guide prior to interviews is a strength of this study. This approach allowed participants time to consider their feelings about events during the pandemic and make decisions about what they wanted to share. It is possible that this reflection led to the non‐disclosure of some experiences that participants did not want to share, but ethically this reinforces the positive nature of this methodological approach. Whilst the aim of qualitative research is not to produce results that are generalisable, the transferability of the findings to individuals with different circumstances or characteristics can be considered (Braun and Clarke 2022). Whilst the sample was small, it was still possible to identify experiences that appeared more universal, such as the loss of meaningful activities, as well as those that seemed more specific to certain circumstances, such as the unique challenges associated with residing in a group‐living environment. It is a limitation of this study that the unique challenges associated with group‐living could not be explored in more depth. Future research may wish to build on this limitation and conduct interviews with a more homogeneous sample comprising individuals who all resided in group‐living environments during the pandemic. Another limitation regarding the transferability of the findings is that the interviewees were living more enriched lives than many people with intellectual disabilities; most had some form of paid or voluntary work, were connected with advocacy organisations and were digitally skilled. As such, these findings may not transfer to individuals who are more socially isolated, or those who would be unable to participate in a one‐to‐one interview. Given these additional layers of inequality, such individuals will have likely faced challenges different in both quality and severity during the COVID‐19 pandemic.

## Author Contributions

**Jodie Rawles:** wrote the first draft, data collection, data analysis, review and editing. **Sue Caton:** conceptualization, wrote the first draft, review and editing. **Dawn Cavanagh:** data collection, data analysis, review and editing. **Chris Hatton:** conceptualization, writing – review and editing. **Richard P. Hastings:** conceptualization, writing – review and editing.

## Conflicts of Interest

The authors declare no conflicts of interest.

## Data Availability

The data that support the findings of this study are available on request from the corresponding author. The data are not publicly available due to privacy or ethical restrictions.
